# Quality Definition in Micro Injection Molding Process by Means of Surface Characterization Parameters

**DOI:** 10.3390/polym14183775

**Published:** 2022-09-09

**Authors:** Vincenzo Bellantone, Rossella Surace, Irene Fassi

**Affiliations:** 1 STIIMA-CNR, Institute of Intelligent Industrial Systems and Technologies for Advanced Manufacturing, Consiglio Nazionale delle Ricerche, Via Lembo 38/F, I-70124 Bari, Italy; 2 STIIMA-CNR, Institute of Intelligent Industrial Systems and Technologies for Advanced Manufacturing, Consiglio Nazionale delle Ricerche, Via Corti 12, I-20133 Milano, Italy

**Keywords:** micro injection molding, DoE, quality index, surfaces characterization

## Abstract

Quality evaluation of micro injection molded products is a complex task, in particular when instruments basing on contact methods are used and issues in measurements could arise due to the contact tool dimension not fitting well with extremely narrow features. Therefore, in these cases, optical methods may be preferred for the evaluation of molded products’ dimensions and surface quality, especially for parts devoted to applications requiring functional purposes. In this context, the present paper proposes the use of surface parameters as a quality index for the evaluation of both the micro injection molding process and the resulting products. To this aim, two experimental procedures were implemented to allow for: (i) the evaluation of the most suitable surface parameters identified in relation to the process parameters; (ii) comparisons of the surface parameters findings with those obtained by classic dimensional quantity via a designed experimental plan (DoE). The results show that the surface parameters, evaluated in critical areas of the components, can ensure reliable estimates for the surface quality of the molded parts and can be preferred in comparison to linear measurements.

## 1. Introduction

The injection molding (IM) process is widely used in several fields, spanning from information technology industry to the automotive, biology, and medical fields. The widespread exploitation of IM is due to its acknowledged mass production capability (low cost–high volumes), along with the high quality and accuracy exhibited by the micro components’ manufacturing.

In the IM process, the molten polymer is injected with a defined speed and pressure into a mold cavity assuming its shape; here, both materials and process parameters play an essential role in producing high quality parts. To this end, the selection of polymeric material is crucial since the type of monomer used directly affects the molding phase [1]. At the same time, reaching the best process parameters setting is a very complex task due to the large number and types of involved factors. Hence, different methods such as the trial and error, analytical, and simulative methods [2,3,4,5] are used to optimize the IM process parameters. Additionally, it must be recognized that the optimized process parameters setting also depends strictly on the variable chosen in the experimental plan as the quality response. Usually, such a variable is selected based on the functionality of the injected parts. So, it can be related to the geometrical features (dimensions or surface roughness) and the material or the mechanical properties (shrinkage, hardness, breaking stress and strain).

Generally, for the optimization of the IM process and products, some quality indices, such as weight or part size [6,7], can neither be easily evaluated nor provide exhaustive characterization. In particular, for injection molded components, the surface of a molded component is not only important for the look of the part but is essential for the evaluation of its functional properties, as well as for sliding or adhesion. From this viewpoint, the characterization of a part’s surface can be used for the description of material processing, thus revealing its thermal history and providing useful information about the process’ performance [8]. Indeed, previous results reported from the authors of [9] show that surface roughness of cavities greatly affects the process as the molding scale is decreased. In particular, it has been demonstrated that by decreasing the depths of thin cavities (200,−100, and 50 µm), complete component filling can be achieved by high surface roughness. In a following in-depth analysis, these results were correlated with wettability, and it was shown that the flow length increases with both mold roughness and the contact angle between the molten polymer and mold surface. The reason for such a phenomenon can be ascribed to the heat transfer and slippage between the polymer melt and the steel mold. In fact, high values of surface roughness and the contact angle between melt and mold materials increase the amount of air trapped among surface asperities, causing a significant reduction in cooling rate and polymer adhesion to the wall, and thus inducing longer flow length [10]. From this perspective, and from the studies of Zhao et al. [11], Sha et al. [12] and Whiteside et al. [13], it can be stated that, generally, for very small cavities, high values of process parameters have a positive effect on the melt flow. Conversely, other studies [14] demonstrated that by setting higher values for process parameters, micro parts quality was affected negatively, the processing time and cost increased, the and mold’s working life was significantly reduced.

When molding product dimensions are decreased, surface metrology offers different techniques for quality and surface characterization [15], such as optical instrument and methods [16] which can be employed in the field of production engineering [17]. Advances in these measurement techniques allow for increased measure precision in a relatively wide area, thus enabling the evaluation of micro molded lens arrays [16,18] or surface roughness [19]. An important requirement in micro IM is the assessment of the replication of the micro-injected parts. To this end, optical methods can be adequately selected depending on the specific micro/nano features characterizing the parts [20]. Furthermore, the definition of the relation linking IM process parameters and surface roughness is also relevant, as it can provide useful insight towards the optimization of process and products. Indeed, as demonstrated in [21], surface roughness can be modified to a certain extent via the control of the molding conditions.

Basing on the reported literature, since molded part surface is strictly related to the IM process parameters, the use of a parameter such as quality control index is assessed and proposed in the present work. In order to accomplish this goal, an experimental approach, implying two case (A and B) studies and based on ANOVA, is proposed. The former case A is used to identify the relation binding molded parts surface and IM parameters. In particular, a surface parameter that returns better product quality is determined by comparing two samples, one produced at the beginning of the whole part production (low quality sample) and the other one manufactured at steady state condition (i.e., a machine working at full capability with optimized process parameters). In contrast, in order to assess the molded parts quality, experiments gathered within case B are performed to implement a comparison between the detected surface parameters with the ones coming from the conventional part length estimates (linear measurement).

## 2. Materials and Methods

### 2.1. Mold Design and Manufacturing

**Figure 1:**
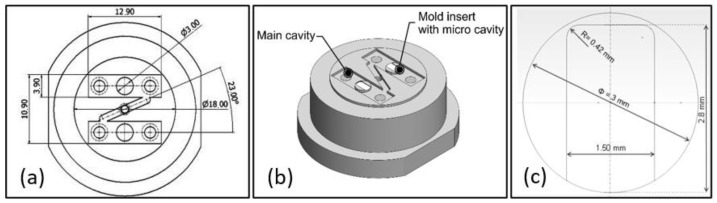
(**a**,**b**) mold and (**c**) insert designs and dimensions; sizes in (**a**,**c**) are reported in mm.

**Figure 2:**
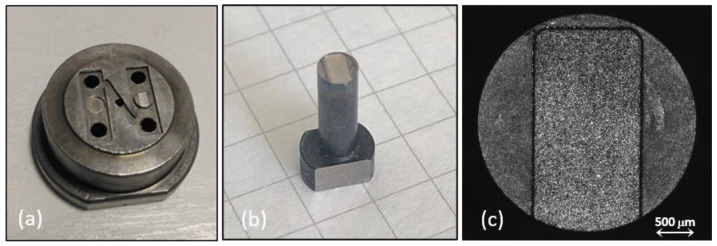
(**a**) Mold, (**b**) insert and (**c**) confocal image of the cavity.

Figure 1 shows (a) the mold, (b) the insert, and (c) the plate cavity designs, while the confocal images of the corresponding realized components are shown in Figure 2. The fabrication of the present mold involves the use of different technologies as micro milling and micro- electric-discharge machining (µEDM). The main part of the cavity was micro-milled by the Evo machine (KERN, Eschenlohe/Murnau, Germany), while the two inserts, characterized by micro-features located in the middle of the part, were manufactured by the SX 200 HP (SARIX, Sant’Antonino, Switzerland) micro-EDM machine [13]. The constraints in mold realization concern the accomplishment of the required high accuracy of micro-sizes and dimensions. Micro-EDM versatility expresses its potential in the possibility of combining different approaches as micro-EDM wire, milling, and sinking, and thus in finding an optimized trade-off between feature accuracy and machining time [14].

The cavity insert has a rectangular geometry with thickness of 100 µm. The filling of the cavity represents a challenge for micro injection molding due to the high aspect ratio [22]. Indeed, it was designed on purpose to test hard settings of the IM process. The reduced gate height at the cavity entry entails a severe condition for the polymer flow, thus hesitation effect occurs and opposes to flow entrance [23]. Conversely, the larger cavity width compared to its depth was chosen in order to minimize boundary condition effects.

### 2.2. Material and Micro-IM Machine

The material used for the proposed study is a commercial polyoxymethylene (POM Ultraform, Basf, Ludwigshafen, Germany). It is a well-known semi-crystalline polymer commonly used in micro-IM applications due to its hardness and stiffness, chemical and mechanical resistance, and low-cost. POM main properties are listed in Table 1.

As suggested by manufacturer, the POM was dried for 3 h at a temperature of 110 °C before micro-IM by means of FormicaPlast 1k machine (DesmaTec, Achim, Germany). The injection unit of this machine is provided of two pistons: a 6-mm piston for the polymer pre-plasticization and a 3-mm piston for the injection, with a maximum injection stroke of 23 mm. The maximum achievable injection pressure and injection rate are 300 MPa and 3.5 cm^3^/s, respectively. Additional details related to the machine are reported in [7].

### 2.3. Optical Measurements for Length and Surface Parts Characterization

The geometrical characterization of micro-components is a challenging task due to difficulties in handling parts of few micron for which contact instruments and standard rules are not applicable. Therefore, optical instruments prove to be more suitable for dimensional evaluation and perform quality characterization. Recent developments in metrology area concern the attempt to include quality characterization by means of optical techniques in the IM process, in order to obtain better precision and more reliable results [24]. In the present work, optical measurements of molded part length and surface measurements are performed via confocal microscope CSM 700 (ZEISS, Milan, Italy) by using a Z-scan acquisitions technique and following the ISO 25178-2 standard, which regulates roughness evaluation from optical topographic measurements.

In order to characterize the surface of the mold and of the molded components, the following roughness parameters have been taken into account: the average roughness (Sa) and the root mean square roughness (Sq), which are the amplitude parameters that illustrate immediately the quality of the evaluated surfaces (i.e., max peak height Sp, max valley depth Sv, and max height Sz, which highlight relevant deviation in the texture characteristics; and skewness Ssk and Kurtosis Sku parameters, which provide a quantitative evaluation of the surface structure and defects). Concerning the latter parameters, Ssk represents the degree of height symmetry (peaks and valley) about a mean plane: if >0, peaks prevail over valleys, while if <0, the opposite is the case. Sku value is a measure of the height deviation from the ideal (bell) shape: if this parameter is >3, the measure is sharper, whereas if it is <3, the deviation is smaller. Generally, when surface heights are normally distributed, Ssk is 0.00 and Sku is 3.00 [25].

**Figure 3:**
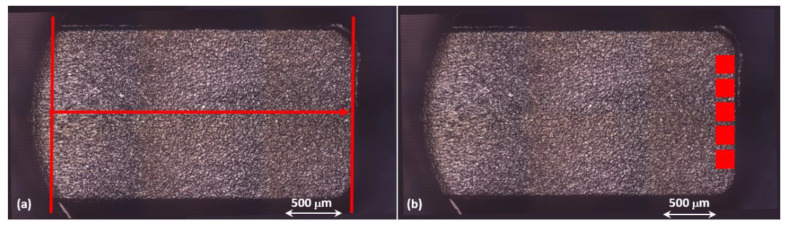
Red lines and areas indicate length (**a**) and the surface (**b**), respectively, considered for the measurements.

Red lines in Figure 3a underline the sample length along the central flow direction used for the measurements and ranging from the gate until the end of the filled section. Figure 3b shows five areas (in red) required for the evaluation of surface parameters, according to the authors of [26]. The location of such areas at the edge of the sample is motivated by the highest sensitivity of this placement to quality variation, since it is farther from the gate and it is also the last filled section (which hardly reproduces the mold surface characteristics). Each area is equivalent to the microscope field of view, 117 µm × 94 µm, at the observation setting (objective lens 100×, z-resolution 0.2 µm). In order to ensure reliability of the measured values, the Chauvenet’s Criterion is implemented, where possible, to individuate outliers. Successively, a Winsorization is applied to the replace outliers due to measurement and/or acquisition errors [27].

### 2.4. Mold Characterization

The mold characterization (Table 2) was carried out before it was assembled on the injection machine by measuring the length of the cavity to be filled and its surface characteristics. The length of injected products is commonly used as dimensional quality index compared to the mold length and, in addition, in this study surface parameter values were also selected as quality indexes for evaluating the process. The parts length and their surface properties will be compared to the mold ones.

## 3. Experimental Plan and Methodology

**Figure 4:**
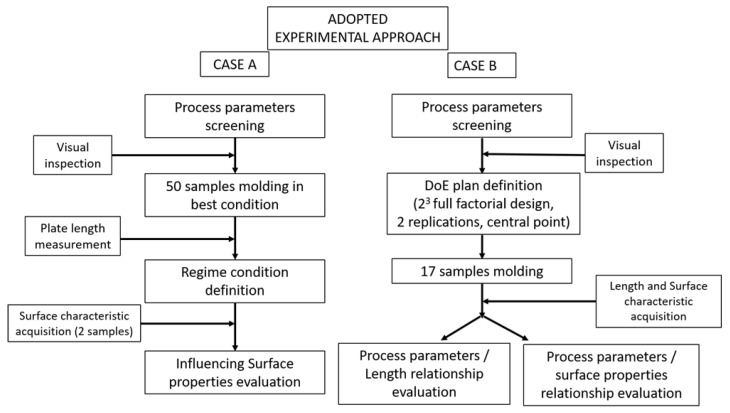
Adopted experimental approach.

The injection molding experimentation has been divided into two steps for cases A and B: the flow chart of the applied procedure is outlined in Figure 4.

Firstly, a screening phase is performed by following the approach described in [9], in order to verify the working technological window of POM. For case A, 50 samples are molded in the best processing condition. Then, length measurements are performed to define the processing regime. Subsequently, surface characteristics of the two samples are acquired. For case B, a Design of Experiment (DoE) [28] is implemented, in order to identify the most effective process parameters both with the length and the surface proprieties as quality indexes.

The process parameters selected for quality evaluation pertaining case A are reported in Table 3: injection speed (Vinj), melt temperature (Tm), and mold temperature (Tmo) are the most important ones which affect the IM process. In particular, it can be noticed that the Vinj presents two values, which are required to set a velocity ramp for the polymer flow. Furthermore, the holding time (th) and pressure (Ph) are fixed, respectively, at 100 Mpa and 3 s; previous studies [9] highlight that the holding phase is not so crucial for micro plate filling. Finally, piston run and cooling time are fixed at 18.2 mm and tc = 5 s, respectively.

For the second step of the experimentation concerning Case B, a design of experiment (DoE) approach is applied to assess the effects of the selected parameters. A two-levels three factors full factorial design is considered, and eight treatments in a randomized sequence are scheduled. Two replications are carried out for a total of sixteen treatments (plus a central point, resulting in seventeen total treatments). The central point was introduced for checking the curvature in the response. The experimental design was chosen by requiring a statistical power of 95%.

Table 4 shows the process parameters and values adopted for the experimental plan of case B. The injection velocity range is very narrow since the filling of this cavity can be achieved only with high velocity, which is required to force the polymer flow in the cavity, overcome the hesitation effect, and prevent the rapid melt freezing. The fixed parameters are set as follows: holding pressure and time = 0 Mpa, and 0 s, respectively; piston run = 7 mm, cooling time = 5 s. As previously mentioned, also in this case, the holding phase has been neglected. Table 5 reports combinations and run order of the experimental plan performed without interruption or material batch change (all treatments belong to the same block) and with one center point. After molding, and for each treatment, the first 10 samples are discarded to stabilize the process. Subsequently, for the same treatment, the following ten samples are collected and three of them, randomly selected, are measured. All of the tests were carried out in a climatic chamber set at 20 °C and RH 50%.

## 4. Results and Discussion

### 4.1. Case A: Evaluating Steady State Condition of the Process

**Figure 5:**
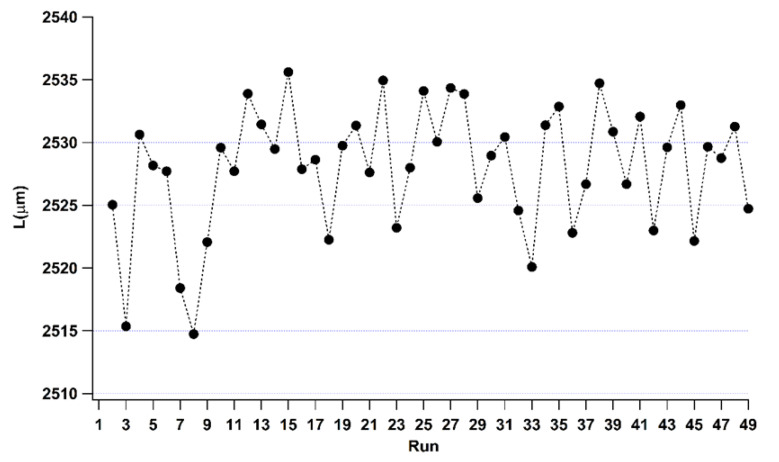
Length values of 48 consecutive molded samples (the first not completed is neglected).

In the IM process, the dimensional characterization of molded components provides information about products quality and process. In this case, the length measurement fulfils this requirement; its target is to achieve the designed mold length. Case A aims at the identification of the threshold defining the steady state condition of the process. Hence, a sample molded before this desired condition (which would be likely of low quality) is selected, along with another one molded when the IM machine is working at its full capability. The differences between the surface parameter values of these two samples are necessary to identify if they can be used as quality indexes. Figure 5 shows the measured length values of 48 consecutive samples: an exception is the first sample, which was incomplete and thus discarded. The obtained values are in the range of 2520–2535 µm: the average length is 2528 µm, with a standard deviation of 5 µm, and few values lying under this range are in the first runs.

In order to evaluate the process stability, Xbar-R chart is used to detect significant process changes 29. These charts display the average data and range, thus allowing one to highlight significant variation of the process and assess the threshold of steady state condition. Since a proper sample size of the subgroups is crucial to detect process shifts correctly, Formula (1), that considers variables estimated by the process and operator constraints, is adopted for sample size:

(1) n=Zα2+Zβ2·σ2D2,

where *n* is the required sample size, *Z_α_*_/2_ and *Z_β_* are the number of standard deviation above zero on a standard normal distribution such that the area in the tail of the distribution are *α*/2 and *β*, respectively; *α* is the type I error probability and is set to 0.0027 for control chart application; *β* is the type II error probability set to 0.1 [29]; *σ* is the standard deviation and *D* is the difference capable of detecting values lying outside the range of the steady state condition. *D* is set to 7.5, lower than ±*σ* range, to ensure a reasonable sensibility to process shifts. By considering the reported values, n results to be equal to 8.

**Figure 6:**
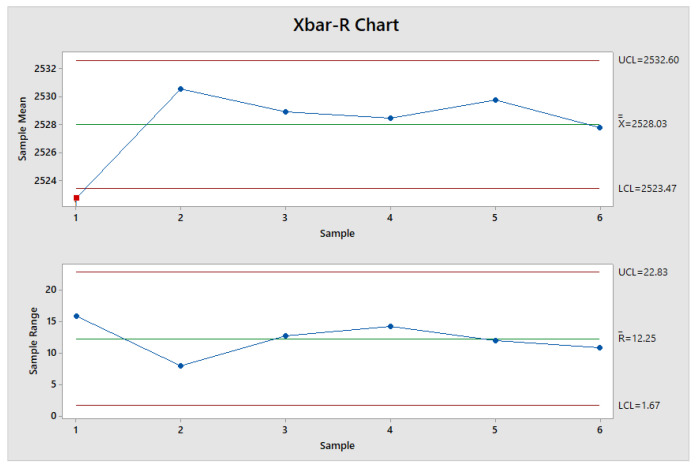
Control charts: Xbar-R chart.

Figure 6 reports the control charts for the measured samples, obtained by Minitab^®^ software, where each point represents a sample size of eight consecutive molded samples. Range chart (lower plot in Figure 6) shows that the process is in control hence the Xbar chart (upper plot in Figure 6) can be considered valid. Moreover, the Xbar displays that the point 1 is out of the lower control limit and it is more than 3 standard deviations away from central line.

As inferable from the control charts analysis and results, the IM process achieves the steady state condition starting from run_9. Figure 5 shows that the sample manufactured in the run_8 is the shortest one produced during the starting production condition (transient condition) and, consequently, it represents the lowest-quality product selected. Therefore, sample of run_8 is then compared with run_36, which is randomly chosen among the samples manufactured during steady state process condition. Finally, samples lengths of run_8 and run_36 are equal to 2514.7 µm and 2522.8 µm, respectively.

### 4.2. Case A: Evaluating Effective Surface Parameters

**Figure 7:**
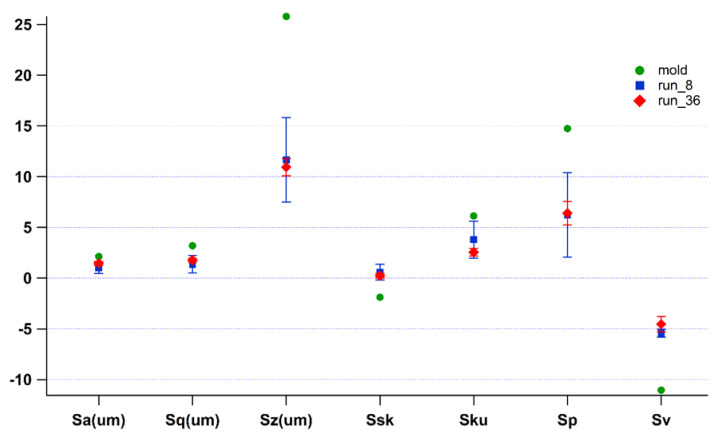
Surface parameters for run_8 (blue squared dots), run_36 (red rhombic dots), and mold (green dots).

High replication of the mold surface in a critical area, as for example the component edge described in Figure 3, guaranties the completion of mold filling and the achievement of the designed thickness. In order to demonstrate that high quality components can be identified by the capability of molded parts to replicate the mold surface, surface parameters need to be evaluated. Figure 7 reports the surface parameters results for the lowest-quality sample (run_8, blue squared dots) and for the steady state one (run_36, red rhombic dots), along with their corresponding standard deviations. Green dots represent the surface parameters evaluated for the mold. As evident from the plot, both samples show that surface values of Sz, Sp, and Sv indicate the presence of noticeable peaks and valleys. Moreover, for both runs, these values are far apart from the corresponding mold values, thus suggesting an independence of the IM process condition stability. This result is due to the difficulties experienced by the molten polymer to fill the micro plates and so replicate the mold surface. Nonetheless, it is worth noticing that the IM process conditions are significantly stressed, as temperature value is at the highest level of material processability range and injection speed value is set higher than the standard injection molding range. Furthermore, surface parameters of run_8 present larger standard deviation than run_36, confirming the variability of the process.

**Figure 8:**
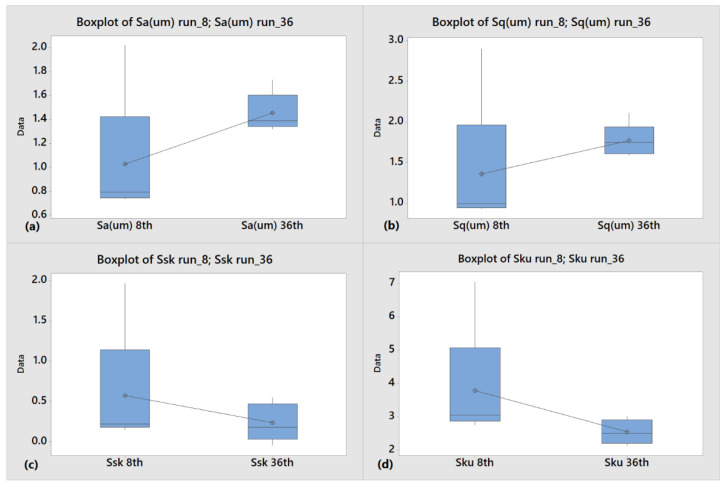
Box plots of (**a**) Sa, (**b**) Sq, (**c**) Sku, and (**d**) Ssk for run_8 and run_36.

Figure 8 shows the box plots for Sa (Figure 8a) and Sq (Figure 8b) parameters of the analyzed samples. These parameters are insensitive to the presence and distributions of peaks and valleys and are ultimately redundant due to their similar trends. Hence, Sa was the only considered parameter. In particular, Sa for run_8 has a lower average value (1.0 µm) than run_36 (1.5 µm) and mold (2.1 µm, Table 2), although run_8 exhibits a wider dispersion. Negative mold value of Ssk (−1.9, Table 2) evidences the predominance of valleys. This result is consistent with the typical appearance induced by micro-EDM machining adopted for mold manufacturing. In addition, this surface parameter presents a wide dispersion and worse value for the sample obtained in an unstable condition (run_8) with respect to the one manufactured in a steady state condition (Figure 8c). A similar behaviour is observed for the dispersion of the Sku parameter (Figure 8d). The value for run_8 is closer to mold one compared to run_36, even if both values are still far from mold one. This occurrence highlights the variability associated with run_8 as the sample surface becomes more defective (thus increasing the Sku value).

These results suggest that surface roughness (the Sa parameter) is an effective index for evaluating quality of micro molded components and replication capability of the process by considering its average value and dispersion.

### 4.3. Case B: Products and Process Quality Evaluation by Means of Dimensional and Surface Parameters

The next step has the aim of identifying the best molded products. This task is accomplished by evaluating surface parameters and comparing the results with the ones obtained by using length component as the main quality index.

#### 4.3.1. Dimensional Quality Index: Part Length

**Figure 9:**
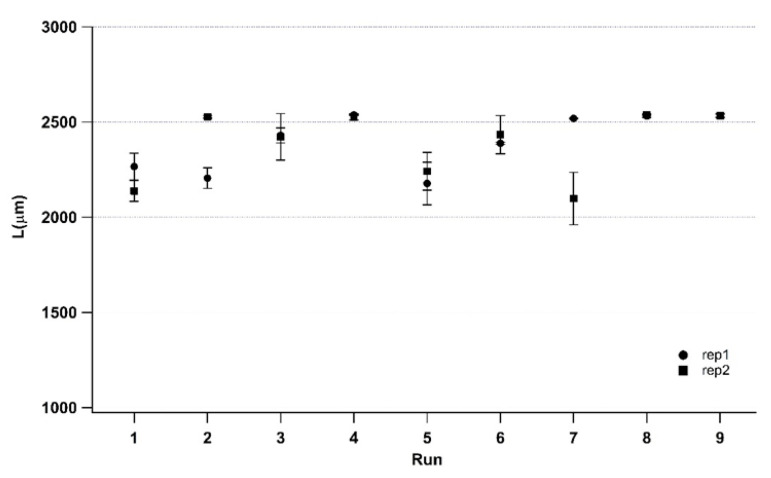
Component lengths and standard deviation of three samples for each treatment of the two replications.

Figure 9 reports the average component lengths pertaining the run order for the two replications. The corresponding standard deviations, performed on three measurements repeated for each treatment, are depicted as vertical lines. The trends of the two replications are generally similar, thus indicating the good repeatability of the process. The standard deviations are similar for each treatment, and this demonstrates a wide range of treatments exhibiting low average length. These results are coherent since, when the process is less capable of achieving the complete mold filling, a larger standard deviation is obtained. Conversely, when high average length is reached, good repeatability is achieved.

**Figure 10:**
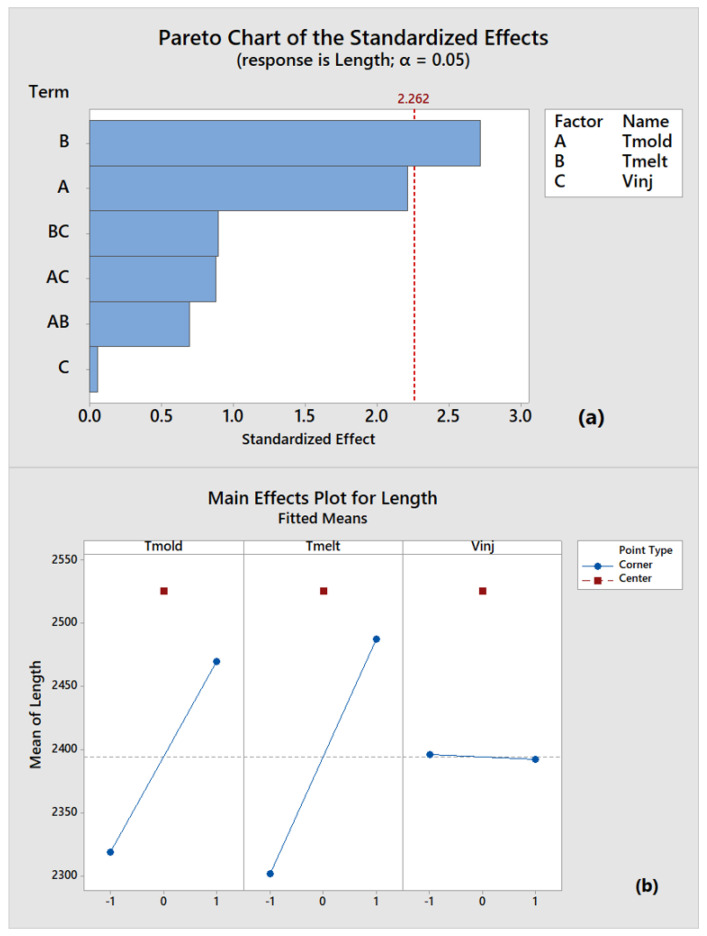
(**a**) Pareto chart and (**b**) main-effect plots of process parameters by considering length as quality index.

The normal distribution and the homogeneity of variances were both verified. Hence, the ANOVA analysis has been properly carried out. Figure 10 shows the Pareto chart of the standardized effects (Figure 10a) and main-effects plots (Figure 10b) for the process parameters involving samples length measurements as the main index. The Pareto diagrams show that only melt temperature exceeds the threshold (red dotted line), beyond which the factors become statistically significant at the chosen α value (0.05). In addition, the mold temperature stops slightly before the threshold, while the injection velocity stops considerably before the first-order parameter interactions. Hence, mold temperature contribution can be regarded as effective (even if with less confidence). The main plots, reported in Figure 10b, indicate how parameter levels (high or low) affect the IM process. The melt and mold temperatures are more effective for cavity filling at higher levels. These results confirmed that high melt temperatures are favourable to polymer flow by reducing its density. Furthermore, high temperatures delay the polymer freezing, which is very rapid in micro-IM of high aspect ratio components due to the high thermal exchange between polymer and cavity surfaces. Finally, the charts show that the central point crosses the upper length value, thus demonstrating an overall non-linear trend.

#### 4.3.2. Surface Quality Index

Considering the results of the box plot shown in Figure 6, the surface roughness parameters appear appropriate as a surface quality index alternative to length measurements for dimensional quality index.

**Figure 11:**
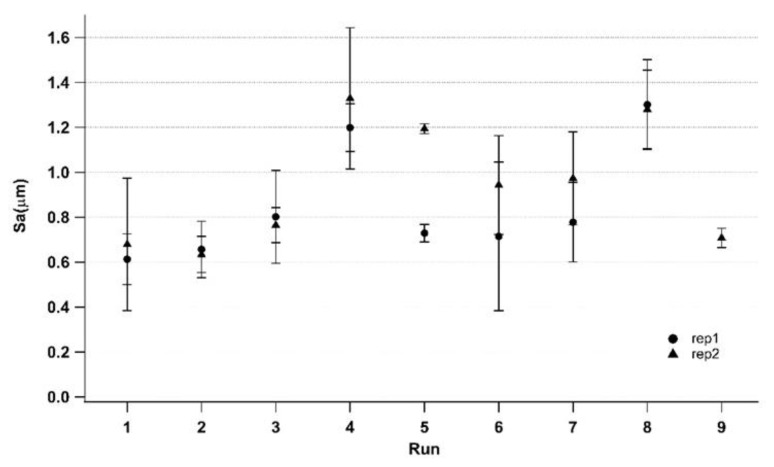
Sa and corresponding standard deviations for each treatment of the two replications.

Figure 11 reports the average surface roughness for the two replications with their corresponding standard deviations. For each treatment, measurements of two samples are performed on five surface areas placed at the edge of the components, as shown in Figure 3. The trends of the two replications are generally similar, confirming the good repeatability of the process, as already witnessed by the previous dimensional characterization. The standard deviations also have close values for each treatment. Generally, a wide range can be appreciated (except for run_5, which also displays a relevant difference between the two replications). The achievement of surface replication between the mold and its part is complicated at the edge of a very thin part due to the cumbersome filling phase. In fact, the highest achieved surface roughness is far from the corresponding mold value (Table 2).

**Figure 12:**
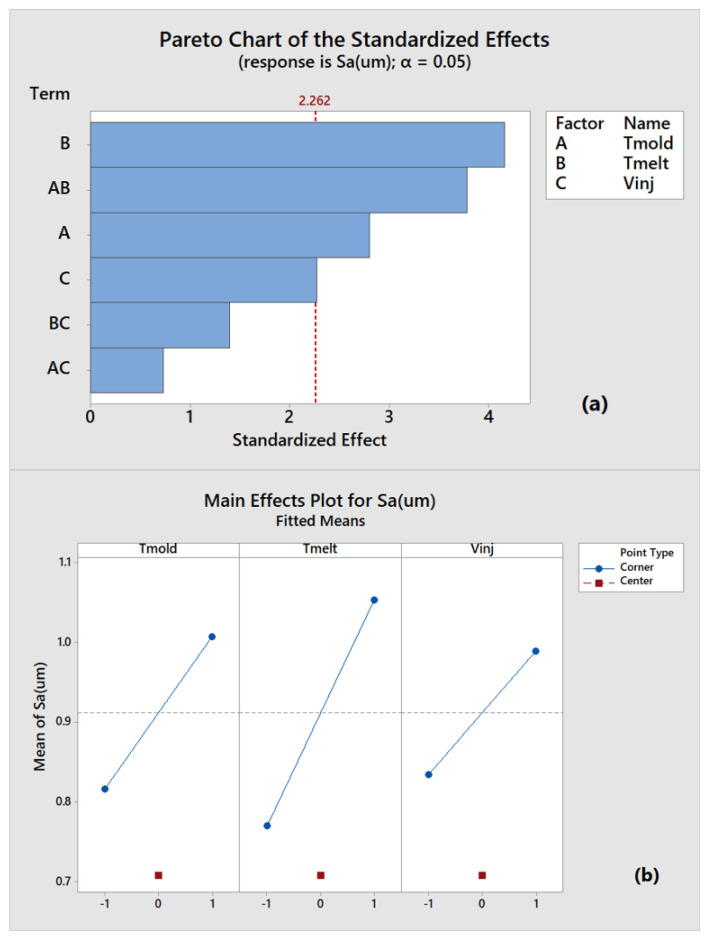
Pareto chart (**a**) and main-effect plots (**b**) of process parameters by Sa.

Due to the compliance with the normal distribution, the homogeneity of variances conditions, and considering previous results (Figure 4), the ANOVA analysis was carried out on the Sa parameter. For the sake of completeness, it should be noted that the Sq parameter is redundant compared to Sa, while Ssk and Sku show a wider standard deviation but similar average value between low-quality samples (run_8) and the one produced in a steady state condition(run_36). Figure 12 shows the Pareto chart of the standardized effects (a) and the main-effect plots (b) for the process parameters considering Sa as the main quality index. Pareto chart indicates that melt and mold temperatures and their interaction are responsible for the statistically significant threshold (red dotted line) at the chosen α value (0.05), and, thus, they are the most effective parameters in the IM process. Therefore, melt temperature confirms the result obtained by analyzing the components length as the most effective parameter on micro cavities molding. However, the Sa analysis also demonstrates the significant role played by mold temperature, as suggested by main-effect plot (Figure 12b). Indeed, this process parameter favours surface covering before melt freezing. The injection velocity falls in correspondence to the statistically significant threshold, and its importance in the process has been already stressed in the initial sections. The central point falls under a lower Sa value, pointing out an overall non-linear trend. The main-effect plots demonstrate the importance of setting high parameters levels for filling micro-mold cavities and achieving satisfying results.

### 4.4. Discussion

The results related to Case A presented in Section 4.2 allow us to identify Sa as the most adequate surface parameter involved in evaluating both the micro-IM process and its products. The most significant process parameters among mold temperature, melt temperature, and injection velocity are analyzed by the ANOVA method. In the analysis, both sample length and surface roughness are used as quality outputs for the experimental plan. The results confirm the paramount importance of temperatures, even if (when length is used as quality index) melt temperature becomes the predominant parameter. In contrast, when surface roughness is chosen as the quality output, the mold temperature results are also significant. This result is consistent since a high mold temperature facilitates the melt fitting to surface mold variations, thus leading to a better surface reproduction. Otherwise, when the length of molded samples is considered as the quality output, the perspective of the process changes to a focus on the mold’s capability to enter a thin cavity for as long as possible. So, a high melt temperature is required to delay melt freezing.

**Figure 13:**
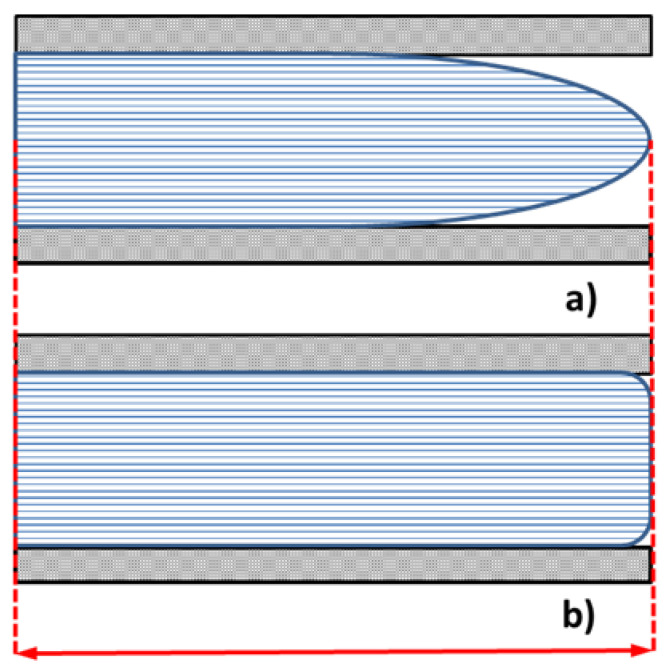
Polymer flow in the mold, at the same length the component is (**a**) incomplete or (**b**) complete.

A remarkable result is that higher length values (Figure 9) do not have counterparts for higher surface values (Figure 11). This means that a higher length value does not guarantee surface roughness replication. Conversely, when higher surface roughness replicability is obtained, higher length can be also evidenced. In fact, as described in Figure 13, a correct component length, equal to mold length, may imply an incomplete filling of the mold and a consequent low-quality part. This result suggests that the use of surface parameters allows for a more precise assessment of the micro injection molding process’ effectiveness. The high-quality surface replication, obtained in the most difficult filling area as the farthest from the gate, ensures the high quality of the entire micro molded sample. Hence, the evaluation of a micro molded component by surface parameters is more advisable in comparison to the evaluation of a single linear dimension.

## 5. Conclusions

This paper investigates the reliability of surface parameters as quality indices for the evaluation of the micro injection molding process and products, involving high aspect ratio components with a thickness of 100 µm. To this end, surface parameters are measured in a critical area and compared to a single linear dimension. A two-step methodology has been proposed to define the effect of surface parameters and process parameters on surface quality indexes. The first analysis demonstrated that the most sensible surface parameter for IM process variation is Sa. Secondly, a full factorial experimental plan has been designed and performed by choosing mold and melt temperatures and injection velocity as the most effective process parameters. The statistical results, analyzed by ANOVA, have been processed by considering both part length and surface roughness Sa as surface quality outputs. The comparison between the two ANOVA analyses shows that both indexes are capable of identifying the same process parameters as effective or high-quality parts. Nonetheless, the use of surface parameters as a quality index provides greater accuracy in part quality control than length, as the best replication of the mold surface in critical areas ensures the best overall quality of the entire component.

## Figures and Tables

**Table 1:** Properties of POM.

Name	Trade Name	Grade	Manufacturer	MVR (cm^3^/10 min)	Density (kg/m^3^)
polyoxymethylene	Ultraform	N2320 003	Basf	7.5	1400

**Table 2:** Mold length and surface characteristics.

	L (µm)	Sa (µm)	Sq (µm)	Sz (µm)	Ssk	Sku	Sp	Sv
**Mold**	2579.4	2.1	3.2	25.8	−1.9	6.1	14.7	−11.1

**Table 3:** Case A: process parameters setting.

Tm (°C)	Tmo (°C)	Vinj (mm/s)	Piston Run (mm)	Ph (Mpa)	th (s)	tc (s)
230	90	150–330	18.2	100	3	5

**Table 4:** Case B: process parameters setting.

Parameter	Description	Low Level −1	Central Point 0	High Level +1
Tmo (°C)	Mold temperature	90	95	100
Tm (°C)	Melt temperature	230	235	240
Vinj (mm/s)	Injection velocity	140–260	150–260	160–260

**Table 5:** Experimental treatments and run order.

StdOrder	RunOrder	CenterPt	Blocks	Tmold	Tmelt	Vinj
15	1	1	1	−1	1	1
9	2	1	1	−1	−1	−1
14	3	1	1	1	−1	1
5	4	1	1	−1	−1	1
6	5	1	1	1	−1	1
16	6	1	1	1	1	1
13	7	1	1	−1	−1	1
8	8	1	1	1	1	1
2	9	1	1	1	−1	−1
3	10	1	1	−1	1	−1
11	11	1	1	−1	1	−1
10	12	1	1	1	−1	−1
17	13	0	1	0	0	0
12	14	1	1	1	1	−1
4	15	1	1	1	1	−1
7	16	1	1	−1	1	1
1	17	1	1	−1	−1	−1

## Data Availability

The data presented in this study are available upon request from the corresponding author.
